# Intra-species recombination among strains of the ampelovirus *Grapevine leafroll-associated virus 4*

**DOI:** 10.1186/s12985-019-1243-4

**Published:** 2019-11-19

**Authors:** Jati Adiputra, Sridhar Jarugula, Rayapati A. Naidu

**Affiliations:** 1 Department of Plant Pathology, Irrigated Agriculture Research and Extension center, Washington State University, Prosser, Washington 99350 USA; 2 Present address, Center for Diagnostic Standards of Agricultural Quarantine, Ministry of Agriculture, Indonesia Agricultural Quarantine Agency, Jakarta, Indonesia

**Keywords:** Grapevine leafroll disease, *Grapevine leafroll-associated virus 4*, *Ampelovirus*

## Abstract

**Background:**

Grapevine leafroll disease is one of the most economically important viral diseases affecting grape production worldwide. *Grapevine leafroll-associated virus 4* (GLRaV-4, genus *Ampelovirus*, family *Closteroviridae*) is one of the six GLRaV species documented in grapevines (*Vitis* spp.). GLRaV-4 is made up of several distinct strains that were previously considered as putative species. Currently known strains of GLRaV-4 stand apart from other GLRaV species in lacking the minor coat protein.

**Methods:**

In this study, the complete genome sequence of three strains of GLRaV-4 from Washington State vineyards was determined using a combination of high-throughput sequencing, Sanger sequencing and RACE. The genome sequence of these three strains was compared with corresponding sequences of GLRaV-4 strains reported from other grapevine-growing regions. Phylogenetic analysis and SimPlot and Recombination Detection Program (RDP) were used to identify putative recombination events among GLRaV-4 strains.

**Results:**

The genome size of GLRaV-4 strain 4 (isolate WAMR-4), strain 5 (isolate WASB-5) and strain 9 (isolate WALA-9) from Washington State vineyards was determined to be 13,824 nucleotides (nt), 13,820 nt, and 13,850 nt, respectively. Multiple sequence alignments showed that a 11-nt sequence (5′-GTAATCTTTTG-3′) towards 5′ terminus of the 5′ non-translated region (NTR) and a 10-nt sequence (5′-ATCCAGGACC-3′) towards 3′ end of the 3′ NTR are conserved among the currently known GLRaV-4 strains. LR-106 isolate of strain 4 and Estellat isolate of strain 6 were identified as recombinants due to putative recombination events involving divergent sequences in the ORF1a from strain 5 and strain Pr.

**Conclusion:**

Genome-wide analyses showed for the first time that recombinantion can occur between distinct strains of GLRaV-4 resulting in the emergence of genetically stable and biologically successful chimeric viruses. Although the origin of recombinant strains of GLRaV-4 remains elusive, intra-species recombination could be playing an important role in shaping genetic diversity and evolution of the virus and modulating the biology and epidemiology of GLRaV-4 strains.

## Background

Grapevine leafroll-associated viruses (GLRaVs, family *Closteroviridae*) represent a group of highly complex and genetically distinct viruses infecting an agriculturally important perennial fruit crop [1]. Among the six distinct species of GLRaVs, GLRaV-1, − 3, − 4, and − 13 belong to the genus *Ampelovirus*, whereas GLRaV-2 and -7 belong, respectively, to the genus *Closterovirus* and genus *Velarivirus* [2]. Thus, the genus *Ampelovirus* contains higher number of GLRaVs compared to other genera in the family *Closteroviridae*. Although all GLRaVs are predominantly disseminated via plant propagation material, grapevine-infecting ampeloviruses are known to be transmitted by mealybugs (Pseudococcidae) and scale insects (Coccidae) in a semi-persistent manner [3]. In contrast, vectors for GLRaV-2 and GLRaV-7 are currently unknown [3]. Nevertheless, GLRaV-7 was shown to be transmitted by the plant parasitic dodder, *Cuscuta reflexa* [4].

GLRaVs in the genus *Ampelovirus* are clustered into two subgroups, based on their phylogenetic divergence and genome size and organization [5, 6]. GLRaV-1, − 3, and − 13, with a large genome size varying between ~ 18.5 and ~ 18.9 kilobases (kb) and encoding nine to twelve open reading frames (ORFs), were clustered under subgroup I. In contrast, GLRaV-4 and its strains with a smaller genome size between ~ 13.6 and ~ 13.8 kb and encoding six ORFs were assigned to subgroup II. Interestingly, currently known strains of GLRaV-4 stand apart from other GLRaV species in lacking the minor coat protein [7]. GLRaV-4 is also unique in that this species is made up of several distinct strains, designated as GLRaV-4 strain − 4, − 5, − 6, − 9, −Pr, and -Car. Recently, a distinct strain of GLRaV-4, designated as GLRaV-4 strain Ob, was described showing close relationship with GLRaV-4 strain Ru [8, 9]. Collectively, all strains of GLRaV-4 are referred to as “grapevine leafroll-associated virus 4-like viruses” or GLRaV-4-LVs [7].

Information on the complete genome sequence and genome organization is available for the different GLRaV-4 strains; namely, GLRaV-4 strain 4 [10], GLRaV-4 strain Pr [11], GLRaV-4 strain Car [12], GLRaV-4 strain 6 [10], GLRaV-4 strain 9 [13] and strain Ob [9]. In contrast, less than full genome sequence is available for GLRaV-4 strain 5 [14]. Within the United States, GLRaV-4 strains − 4, − 5, − 6, − 9, and -Car were reported from California [10, 12, 15, 16] and GLRaV-4 strain 5 from New York [14]. In Washington State, GLRaV-4 strains − 4, − 5, and − 9 were documented in wine grape (*Vitis vinifera*) and juice grape (*V. labrusca* ‘Concord’) cultivars [17–20].

In this study, the full genome sequence was determined for GLRaV-4 strains − 4, − 5, and − 9 from Washington vineyards to examine their genome organization in comparison with GLRaV-4 strains reported from other grapevine-growing regions. Further, phylogenetic and recombination analyses of currently known GLRaV-4 strains showed evidence for recombination events in some isolates of GLRaV-4 strains. The results presented here indicates the occurrence of recombination between distinct strains of GLRaV-4 and such intraspecies recombination can play a role in shaping genetic diversity of the virus and influence the biology and epidemiology of GLRaV-4 strains.

## Methods

### Virus isolates

Isolates of GLRaV-4 strains 4 (WAMR-4) and 5 (WASB-5) were obtained, respectively, from wine grape cultivars Merlot and Sauvignon Blanc planted in two separate commercial vineyards. An isolate of strain 9 (WALA-9) was obtained from the cultivar Lagrein in a varietal collection maintained by a grapevine nursery. The red-berried cultivars Merlot and Lagrein displayed interveinal reddening of leaves with ‘green veins’ and the white-berried cultivar Sauvignon Blanc exhibited mild yellowing of leaves. The presence of GLRaV-4 strains in individual vines was confirmed by single tube-one step RT-PCR assays [21, 22]. Primer pairs LR4/5-Unl370/F and LR4/5-Unl370/R [23] and LR9/F and LR9/R [24] were used for the detection of GLRaV-4 strains 4, 5, and 9, respectively.

### High-throughput sequencing

Spectrum™ Plant Total RNA kit (Sigma-Aldrich, St. Louis, MO) was used to isolate hiqh quality total RNA from petiole samples and cambial scrapings of individual grapevines tested positive for GLRaV-4 strains mentioned above. The quantity and quality of total RNA in each preparation was measured using a Nanodrop 2000c (Thermofisher scientific, Grand Island, NY). The RNA integrity was measured using 2100 Bioanalyzer system (Agilent Technologies, SantaClara, CA). The RNA with a RNA integrity number (RIN) higher than 7.0 was sent to Huntsman Cancer Institute, Salt Lake City, USA, for ribosomal RNA (rRNA) depletion, library construction, and sequencing on a Illumina Hi-Seq 2500 platform (Illumina, Inc., San Diego, CA) in the 125 base-length paired-end mode. Raw sequence reads from each sample were individually imported into CLC Genomics Workbench version 8.0 (Qiagen Sciences Inc., Germantown, MD) and trimmed to remove adapter sequence and analyzed for quality (limit = 0.05) and ambiguity (allowing 2 nucleotide mismatches). Reads matching with rRNA sequences, bacterial and host genomes were filtered from the trimmed paired-end reads and assembled de novo using CLC Genomics Workbench 8.0 with default parameters to produce a pool of contigs. The contigs were subsequently annotated using BLASTX against the non-redundant plant virus database as a reference available from GenBank (http://www.ncbi.nlm.nih.gov/blast). Internal gaps in viral genome sequence were filled by reverse transcription-polymerase chain reaction (RT-PCR) using species-specific primers designed based on high-throughput sequencing (HTS) data and reference sequence corresponding to strain 4, 5, and 9 obtained from GenBank. Total RNA preparations originally utilized for HTS were subsequently used for cDNA synthesis with random hexamer primers (New England Biolab, Ipswich, MA). PCR amplification was carried out using species-specific primers and the amplicons cloned and sequenced from both orientations using Sanger sequencing. The derived sequences together with the de novo assembled contigs were manually edited and assembled to generate the near complete genome sequence for GLRaV-4 strains 4, 5, and 9.

### Determination of 5′ and 3′ terminal sequences

The 5′ terminal sequence for GLRaV-4 strain 4 and strain 5 was determined using a commercially available rapid amplification of cDNA ends [RACE] system (Version 2.0, ThermoFisher Scientific, Grand Island, NY), as described in Donda et al. [25]. For additional confirmation of the 5′ terminal nucleotide, dA-tailing method was used as described earlier by Donda et al. [25]. The 5′ terminal sequence for GLRaV-4 strain 9 was determined using FirstChoice® RLM-RACE Kit (Ambion, Austin, TX, USA), according to the manufacturer’s instructions, since the 5′ RACE system Version 2.0 mentioned above was not successful. To determine the 3′ terminal sequence of GLRaV-4 strains, A-tailing of the 3′ end of viral RNA using Poly(A) polymerase (New England Biolab, Ipswich, MA) was employed as described earlier [26]. Subsequently, C-tailing of the 3′ end of viral RNA was used employing Poly(U) polymerase (New England Biolab, Ipswich, MA) for resolving ambiguity that may occur because of the presence of “A” as the 3′-terminal nucleotide. A list of primers used in these methods is provided in Additional file Table S1.

### Sequence comparison and phylogenetic analysis

The accession numbers of GLRaV-4 strains − 4, − 5, and − 9 generated in this study and extracted from GenBank are listed in Table 1. Multiple alignment of nucleotide (nt) and amino acid (aa) sequences and pairwise sequence identities were carried out using the Muscle program [27] embedded in Molecular Evolutionary Genetics Analysis software (MEGA7) [28]. Distribution of genetic divergence across the genome was analyzed by comparing the GLRaV-4 strains from Washington with corresponding reference sequences obtained from GenBank using SimPlot (Version 3.5.1) [29]. The nucleotide similarities shown in SimPlot analysis were generated by using Kimura 2 parameter distance model with a 200-nt sliding window moved along the sequence in 20-nt steps. Phylogenetic analysis of GLRaV-4 strains from Washington and those obtained from public databases was inferred by Maximum-likelihood method [30] with genetic distances estimated using the best fit nucleotide substitution models identitified in MEGA7. Bootstrap support values based on 1000 replicates were used to determine robustness of the phylogenetic grouping.

**Table 1:** List and identifiers of *Grapevine leafroll-associated virus* 4 strains used in this study. The genome size and length of non-translated regions are shown as nt and open reading frames (ORFs) are shown as aa. Asterisk indicate partial sequence at the 5′-terminus of the virus genome

Strain	Isolate	Accession	Source	genome	5′ NTR	ORF1a	ORF1b	p5	HSP70	p60	CP	p23	3′ NTR
		number		nt	nt	aa	aa	aa	aa	aa	aa	aa	nt
Strain 4	WAMR4	MF669483.1	WA, USA	13,824	215	2344	517	46	533	539	272	207	128
	LR106	FJ467503.1	CA, USA	13,830	216	2345	517	46	533	539	272	207	129
Strain 5	WASB5	MF669481.1	WA, USA	13,820	215	2378	517	46	533	539	269	207	129
	3138–03	JX559639.1	Canada	13,823	217	2378	517	46	533	539	269	207	130
	TRAJ1-BR	KX828702.1	Brazil	13,823	217	2378	517	46	533	539	269	207	130
	Y217	FR822696.2	NY, USA	13,384	82*	2241*	517	46	533	539	269	207	129
Strain 6	Estellat	FJ467504.1	CA, USA	13,807	215	2378	517	46	572	539	269	207	130
Strain 9	WALA9	MF669482.1	WA, USA	13,850	215	2355	517	46	574	539	268	207	125
	Man086	KJ810572.2	Spain	13,858	218	2355	517	46	574	539	268	207	127
Strain Car	Carnelian	FJ907331.1	CA, USA	13,626	214	2287	516	46	534	539	267	207	132
Strain Pr	Pr	AM182328.4	Greece	13,696	213	2294	517	46	533	539	273	207	128
Strain Ob	Ob	KP313764.1	Switzerland	12,849	37*	2076	526	46	581	546	306	207	131

### Recombination analysis

Genome sequences of GLRaV-4 strains were examined for potential recombination events, localization of recombination breakpoints and likely parental sequences using the Recombination Detection Program (RDP) version RDP4.94 with default settings [31]. The RDP software includes a suite of eight recombination-detecting algorithms (see reference [31] for citation of these algorithms) representing the three different types of methods namely, phylogenetic (BOOTSCAN, RDP, and SISCAN), substitution (GENECONV, MAXCHI, CHIMAERA, and LARD) and distance comparison (PHYLPRO) methods to generate evidence of recombination. Using a Bonferroni corrected P-value cut-off of ⩽ 0.05, recombinant sites identified with four or more of the eight algorithms in the RDP were considered ‘significant and clear recombination events’ and recombination events identified by three or fewer programs were considered as ‘tentative recombination events.’ The beginning and end of breakpoints identified with RDP software were used to define putative recombinant sequences that were validated by examination of phylogenetic discordance and pairwise sequence identity. The topologies of phylogegentic trees generated for each recombinant segment were compared to the tree topology obtained from the non-recombinant regions of the virus genomes to examine relationships between the recombinant isolates and other GLRaV-4 strains

## Results

### Genome sequence analysis of three strains of GLRaV-4 from Washington vineyards

After quality trimming, Illumina sequencing generated 29,859,206 paired-end 125 base-length reads from cv. Merlot, 32,073,592 reads from cv. Sauvignon Blanc and 34,512,018 reads from cv. Lagrein. Among these clean reads, 1,396,792 reads (4.68%) from Merlot, 958,285 reads (2.99%) from Sauvignon Blanc and 522,233 reads (1.51%) from Lagrein mapped to reference virus and viroid databases in BLASTX analyses. Reads from each sample were individually assembled de novo to produce a pool of contigs from which those aligning with the genome sequence of GLRaV-4 strains available in GenBank (Table 1) were subsequently used for downstram analyses described below. Contigs corresponding to other viruses and viroids obtained from the three cultivars were not presented (data not shown), since it is outside the scope of this study.

#### GLRaV-4 strain 4 (isolate WAMR-4)

A total of 262,542 quality-trimmed Illumina reads from cv. Merlot formed a single contig of 13,034 nt which aligned with GLRaV-4 strain 4 reported from California (accession no. FJ467503) with approximately 94% genome coverage [10]. After confirming the 5′ and 3′ terminal sequences of the virus genome by RACE and filling the gaps and low coverage regions of the genome as needed by Sanger sequencing of amplicons using species-specific primers (Additional file 1: Table S1), the full-length genome was determined to be 13,824 nt in size (accession no. MF669483). The genome of WAMR-4 isolate was smaller by 6 nt compared to 13,830 nt genome of LR-106 isolate**.** The genome of these two isolates shared 93.2% nucleotide sequence identity, indicating that they are more closely related to each other than to other strains of GLRaV-4 (Additional file 1: Table S2).

#### GLRaV-4 strain 5 (isolate WASB-5)

A total of 349,257 quality-trimmed Illumina reads obtained from cv. Sauvignon Blanc formed a single contig of 13,716 nt, which aligned with GLRaV-4 strain 5 isolates from GenBank. After confirming the 5′ and 3′ terminal sequences and filling the gaps and low coverage regions, the full-length genome was determined to be 13,820 nt in size (accession no**.** MF669481)**.** Previously, the genome sequence of GLRaV-4 strain 5 was reported from Canada (isolate 3138–03, accession no. JX559639**)** and Brazil (isolate TRAJ1-BR, accession no. KX828702) with 13,823 nt in size and from New York (accession no. FR822696) with 13,384 nt in size. However, it should be noted that the exact 5′ terminal genome sequence for isolates from Canada, Brazil and New York was not determined by RACE. Nevertheless**,** the WASB-5 isolate shared approximately 93% nucleotide sequence identity with corresponding sequence of GLRaV-4 strain 5 from Canada, Brazil and New York (Additional file 1: Table S2**),** suggesting that they are genetically related isolates of GLRaV-4 strain 5.

#### GLRaV-4 strain 9 (isolate WALA-9)

The 341,347 quality-trimmed Illumina reads specific to GLRaV-4 strain 9 obtained from cv. Lagrein formed a single contig of 13,816 nt, which aligned with GLRaV-4 strain 9 reported from Spain (accession no. KJ810572). After confirming the 5′ and 3′ terminal sequences and filling the gaps and low coverage regions**,** the full-length genome was determined to be 13,850 nt in size (accession no. MF669482). However, the genome size of WALA-9 isolate was smaller by 8 nt compared to 13,858 nt genome size of GLRaV-4 strain 9 isolate Man086 reported from Spain [13]. Both isolates shared 94% nucleotide sequence identity (Additional file 1: Table S2), indicating that they are closely related to each other than to other strains of GLRaV-4. Thus, WALA-9 isolate represents a new variant of GLRaV-4 strain 9 with similar genome organization between the two isolates.

### Comparative genome organization of three strains of GLRaV-4 from Washington vineyards

**Fig. 1:**
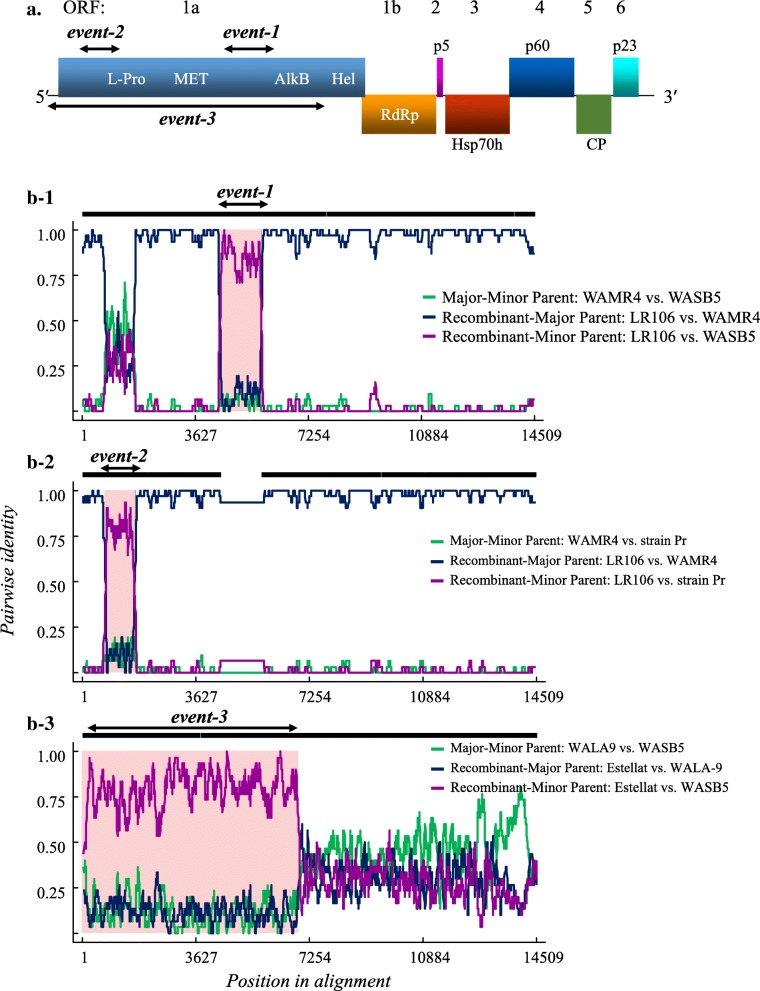
Analysis of recombination events in the genome of GLRaV-4 strains. (**a**) Graphical representation (not drawn to scale) of the generalized genome map of GLRaV-4. Individual open reading frames (ORFs) are shown as boxes with associated protein designations used for closteroviruses [2] and numbered 1 to 6 above the diagram. Abbreviations of ORFs: *L-Pro*, papain-like leader protease; *MET*, methyltransferase domain; *HEL*, RNA helicase domain; *AlkB*, the AlkB domain; *RdRp*, RNA-dependent RNA polymerase; p5, 5 kDa protein; *Hsp70h*, heat shock protein 70 homolog; *CP*, coat protein; p23, 23 kDa protein. Lines at the genome extremities represent non-translated regions. **b** Putative recombinant events in isolates LR106 and Estellat. (B-1) and (B-2) represent, respectively, recombination event-1 (nt 4105–5240) and event-2 (nt 627–1551) in ORF1a of the LR106 isolate and (B-3) represents recombinant event (nt 1–6312) in the genome of the Estellat isolate identified by the RDP. The X-axis indicates the nucleotide position in the alignment and the Y-axis shows informative nucleotide pairwise identity between parental and recombinant isolates. The color key of the parental isolates is shown next to the plots

The genome of GLRaV-4 strains 4, 5, and 9 possesses two large gene modules, similar to other viruses in the family *Closteroviridae* (Fig. 1a) [1, 2]. The replication gene module (RGB), located towards 5′ end of the viral genome, consists of ORF 1a and ORF1b and occupies bulk of the virus genome of all three GLRaV-4 strains. ORF1a encoding a polyprotein of ~ 260 kDa contained signature domains conserved in all closteroviruses [1, 2, 7, 10–13]. These domains are arranged in the polyprotein from N-terminus to C-terminus as follows: a papain-like leader protease (L-Pro) with conserved catalytic residues cysteine (C^444^) and histidine (H^487^) and a predicted cleavage site after glycine (G^504^) [11, 32], a methyltransferase (MET, Pfam 01660, Pfam database 27.0) [33] and helicase (HEL, Pfam 01443). Similar to other ampeloviruses, an AlkB domain (Pfam 03171), belonging to 2OG-Fe(II) oxygenase superfamily [34]**,** was present between MET and HEL domains in the polyprotein and contained characteristic ‘core domain’ with conserved motifs described earlier [25].

Similar to published reports, eight nucleotide sequence (5′…AUGUUUAG…3′) overlaps between ORF1a & b and is highly conserved among the GLRaV-4 strains sequenced to date [7]. The conserved sequence upstream to the stop codon (UAG) for ORF1a is presumably involved in a + 1 ribosomal frameshifting mechanism similar to other closteroviruses [35–39]. The processed product of the large polyprotein corresponding to ORF1b region would potentially yield an estimated 58 kDa protein and contains all eight conserved motifs of the RNA-dependent RNA polymerase (RdRp, pfam 00978) reported in positive-strand RNA viruses [40].

The remaining five ORFs, located downstream of the RGB, form a second gene module and sequentially encode for p5, heat shock protein 70 homologue (HSP70h), heat shock protein 90 homologue (HSP90h or p60), coat protein (CP) and p23. Like in all GLRaV-4 strains, the minor CP (CPm) is absent in GLRaV-4 strains 4, 5, and 9 reported in this study. The C-terminal portion of the p60 protein encoded by all three strains, contains a conserved arginine (R435) and aspartic acid (D472) residues, suggesting the presence of CP-homologous domain in the p60 [41, 42]. The proteins encoded by ORFs 2 to 6 showed characteristics similar to the corresponding proteins of GLRaV-4 strains reported earlier [9–14]. Based on the current understanding of the molecular biology of *Beet yellows virus* (BYV, [43, 44]), *Citrus tristeza virus* (CTV, [45]) and other grapevine leafroll viruses [25, 26], it is likely that ORFs 2 to 6 are expressed from a subset of 3′ coterminal subgenomic RNAs (sgRNAs). Similar to these closteroviruses, each of the sgRNAs encoded by GLRaV-4 strains 4, 5, and 9, except the 3′-most sgRNA coding for p23, is technically polycistronic, but functionally serving as a monocistronic mRNA expressing the 5′-most ORF from individual sgRNAs. In analogy with BYV and CTV, proteins encoded by ORFs 2 to 6 of the three strains of GLRaV-4 are likely multifunctional and responsible for various functions in the virus life cycle, such as intercellular transport, virion assembly and silencing suppression [46–49].

**Fig. 2:**
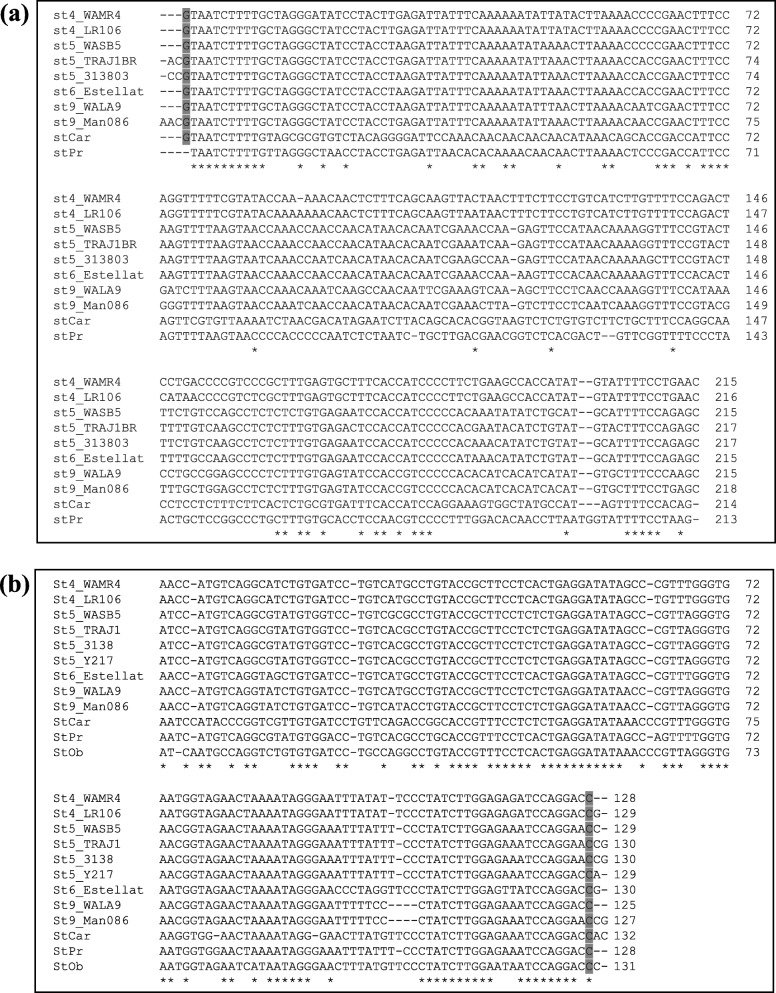
Multiple sequence alignment of the (**a**) 5′ and (**b**) 3′ nontranslated regions of GLRaV-4 strains. Asterisk (*) indicates conserved residues. The conserved nt at the 5' and 3' ends is highlighted. The alignment was adjusted manually and gaps (shown as '-') introduced for optimal alignment of sequences

The 5′ and 3′ NTRs of GLRaV-4 strains 4, 5, and 9 were determined by RACE. The results indicated that all three strains have a 11-nt sequence (5′-GTAATCTTTTG-3′) highly conserved at the 5′ terminus of the genome (Fig. 2a). In multiple sequence alignments, this 11-nt sequence was observed in the 5′ NTR of GLRaV-4 strains 4, 5, 6, 9 and Car. However, two to three extra nts were present upstream of this 11-nt conserved sequence in the 5′ NTR of GLRaV-4 strains 5 and 9 and one nt short in the conserved sequence in strain Pr. The 3′ NTR of GLRaV-4 strains 4, 5, and 9 sequenced in this study contain a 10-nt conserved sequence (5′-ATCCAGGACC-3′) towards the 3′-terminus (Fig. 2b). In multiple sequence alignments, this 10-nt sequence was conserved (except 1 nt) in the 3′ NTR of GLRaV-4 strains sequenced previously, with some of them having one to two additional nts downstream of this conserved sequence [10–13]. Although the exact terminal nucleotide at the 5′ and 3′ end needs to be confirmed for some GLRaV-4 strains, the above observations suggest that GLRaV-4 strains contain a conserved guanidine (G) and cytosine (C) residues, respectively, at the 5′ and 3′ end of their genomes.

### Genome wide sequence divergence

To understand the distribution of sequence divergence across the genome, a SimPlot analysis was carried out by using the complete or near complete genome sequences of GLRaV-4 isolates (Fig. 1b and Additional file 2: Figure S1). The strain 5 isolate Y217 from NewYork and strain Ob isolate were not included in the analysis due to incomplete sequence towards the 5′-terminus. The SimPlot analysis showed that the sequence divergence in WASB-5 and WALA-9 isolates was spread across the entire genome when compared with corresponding strain 5 and strain 9 sequences from GenBank (Additional file 2: Figure S1). In contrast, the SimPlot graph with strain 4 isolate WAMR-4 as query sequence showed abrupt changes in two regions of ORF1a when compared with corresponding regions of strain 4 isolate LR-106 (Fig. 1.B-1 & B-2; Additional file 2: Figure S1.B-1 and Additional file 2: Figure S1.B-2). This was further supported in ORF-by-ORF comparisons between WAMR-4 and LR-106 isolates, where ORF1a showed only 82% aa sequence identity and other ORFs showed greater than 96% aa sequence identity (Additional file 1: Table S2). The two regions in ORF1a corresponding to nt 4105–5240 and nt 627–1551 in the genome of LR106 isolate showed, respectively, 38 and 48% aa sequence identity with corresponding sequence in WAMR-4 isolate (Additional file 2: Table S3). To ascertain that these sequence differences were not due to errors during HTS sequence assembly, the two genomic regions in the WAMR-4 isolate were amplified by RT-PCR using primers designed based on the conserved regions flanking the variable regions (Additional file 1: Table S1). Sequence analysis of the cloned amplicons confirmed the sequence differences observed in the ORF1a of LR106 and WAMR-4 isolates of strain 4. Interestingly, SimPlot analysis of the genome sequence of GLRaV-4 strains also showed abrupt change between the 5′ and 3′ half of the Estellat isolate of strain 6 relative to other strains (Fig. 1.B-3; Additional file 2: Figure S1.B-3). The 5′ half of the Estellat isolate showed high sequence identity with isolates of strain 5 and the 3′ half is relatively distinct from all GLRaV-4 strains (described below). These results indicated possible occurrence of recombination events between GLRaV-4 strains during their evolution and diversification.

### Evidence that some isolates of GLRaV-4 are recombinants

**Fig. 3:**
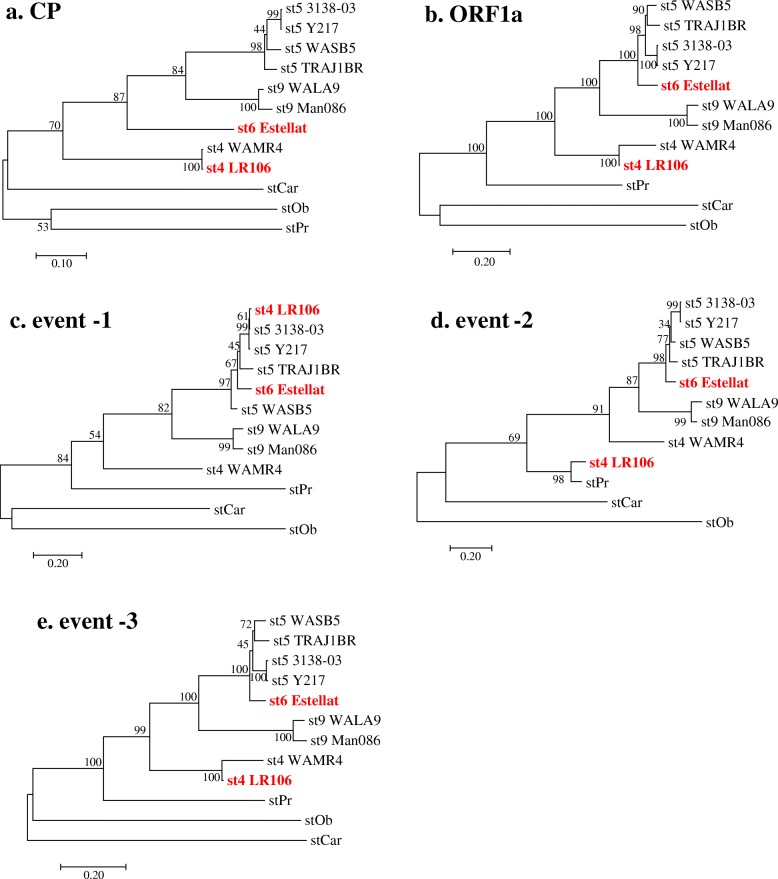
Phylogenetic evidence for recombination among GLRaV-4 strains. Nucleotide sequence corresponding to (**a**) the CP, (**b**) ORF1a, recombinant regions identified for putative (**c**) event-1 (nt 4105–5240) and (**d**) event-2 (nt 627–1551) in ORF1a of the LR106 isolate, and (**e**) event-3 (nt 1–6312) in the Estellat isolate were used for constructing the Maximum-likelihood method-besed trees with 1000 replicates, using the MEGA 7 software. Recombinant isolates showing phylogenetic discordance are indicated in red color. Refer to Fig. 1b and Table 2 for details of putative recombinant event-1, event-2 and event3

A recombination analysis was carried out using the RDP package to confirm recombination signals among isolates of GLRaV-4 strains observed in SimPlot analysis (Fig. 1b). For this purpose, the complete genome sequences of the nine GLRaV-4 strains available from GenBank and sequences of GLRaV-4 strain 4, 5, and 9 generated in the current study were subjected to recombination analysis. The recombinant isolates and their potential ‘parental sequences’ are listed in Table 2 and shown in Fig. 1b. Two putative recombination events were detected in LR106 isolate of strain 4 (accession number FJ467503.1) and one event in Estellat isolate of strain 6 (accession number FJ467504.1) in all eight recombination-detecting algorithms implemented by the RDP with significant statistical support (Table 2). The sequence between nt 4105 and nt 5240 in the genome of LR-106 isolate was identified as a putative recombinant sequence involving strain 4 isolate WAMR-4 as the major parent and strain 5 isolates WASB-5 and TRAJ1-BR and strain 6 isolate Estellat as minor parents (Table 2, Fig. 1B-1). This 1136 nt fragment of the LR106 isolate showed 99% sequence identity with corresponding sequence in isolates of strain 5 from Canada and NY and 88–90% identity with corresponding sequence in isolates of strain 5 from WA and Brazil and in strain 6 isolate Estellat from CA (Additional file 1: Table S3). Similarly, sequence between nt 627 and nt 1551 of the LR106 isolate was identified as the second putative recombinant sequence with strain 4 isolate WAMR-4 from WA and strain Pr from Greece as the potential major and minor parental sequences, respectively (Table 2, Fig. 1B-2). This 925 nt fragment from LR106 isolate showed 89% nucleotide sequence identity with corresponding sequence in strain Pr and less than 50% sequence identity with other strains (Additional file 1: Table S3). These results suggest that the LR106 isolate of strain 4 evolved through at least two recombination events in the ORF1a. The RDP analysis also identified the Estellat isolate of strain 6 as a recombinant (Table 2, Fig. 1B-3). Similar to SimPlot analysis, RDP analysis indicated a recombination break point at nt 6312, approximately in the middle of the genome of Estallat isolate. The sequence upstream and downstream to the breakpoint position were compared with corresponding sequences of other GLRaV-4 isolates (Additional file 1: Table S3). The sequence upstream of the breakpoint between nt 1 and nt 6311 of the virus genome shared 89–90% nt sequence identity with corresponding sequence of strain 5 isolates. In contrast, the sequence downstream of the breakpoint between nt 6312 and nt 13,807 shared a maximum of 72% nucleotide identity with other strains of GLRaV-4. These results suggest that the Estellat isolate of strain 6 evolved through at least one major recombination event.

**Table 2:** Predicted recombination events in GLRaV-4 strains^a^

Predicted Recombinant isolate	Recombination Event*	Predicted breakpoint positions in recombinant		Putative Parental Isolates**		Detection method***							
		Begin	End	Minor Parent	Major Parent	RDP	GENE CONV	Bootscan	Maxchi	Chimaera	SiSscan	LARD	3Seq
St 4_LR106	event-1	4105	5240	St 5_WASB-5	St 4_WAMR-4	5.2 × 10^−265^	1.4 × 10^− 236^	3.8 × 10^− 251^	2.9 × 10^−50^	2.7 × 10^−47^	5.1 × 10^−50^	3.3 × 10^− 316^	2.4 × 10^−13^
				St 6_Estellat									
				St 5_TRAJ1-BR									
St 4_LR106	event-2	627	1551	St Pr	St 4_WAMR-4	2.0 × 10^− 228^	5.4 × 10^− 173^	6.3 × 10^−218^	1.2 × 10^−41^	4.4 × 10^−07^	2.4 × 10^−43^	3.3 × 10^− 316^	2.7 × 10^− 13^
St 6_Estellat	event-3	13,807	6312	St 5_3138–03	Unknown (St 9_Man086)	1.7 × 10^− 109^	1.1 × 10^−88^	9.9 × 10^− 112^	1.3 × 10^−59^	4.2 × 10^−03^	4.3 × 10^−98^	2.4 × 10^− 243^	1.2 × 10^− 13^
				St 5_TRAJ1-BR	Unknown (St 9_WALA-9)								
				WASB-5									

^a^Recombinant and parental isolates and nucleotide position of breakpoints in recombinant isolates LR106 and Estellat are listed. The source of isolates of different GLRaV-4 strains are listed in Table 1. The recombination detection program software package (31) used for the detection of putative recombination events and the corresponding average *P*-values for each event are shown. ^*^See Fig. 1 for details. ^**^ Minor and major parents are the isolates predicted to contribute smaller and larger sequence fragments, respectively. ^***^Detection methods are cited in reference [31]

### Phylogenetic evidence for recombination among GLRaV-4 strains

Since recombination is known to affect the inferred phylogeny, phylogenetic trees were constructed using nt sequence of the ORF1a and the CP of GLRaV-4 strains and compared with trees generated using sequences involved in three putative recombination events (Fig. 3). The Maximum-likelihood analysis showed segregation of currently known GLRaV-4 strains into seven groups based on the CP gene-based phylogeny (Fig. 3a). These distinct groups were identified as strain 4, strain 5, strain 6, strain 9, strain Car, strain Pr, and strain Ob. The three WA isolates, WAMR-4, WASB-5 and WALA-9 clustered, respectively, with strain 4, strain 5, and strain 9 isolates. Phylogenetic analysis of putative recombinant sequences (Fig. 3b-e) indicated discordant relationships between GLRaV-4 strains, with Estallat isolate of strain 6 and LR-106 isolate of strain 4 showing different topological positions depending on the putative recombinant sequence within individual strains. The LR-106 isolate most closely aligned with WAMR4 isolate of strain 4 based on the complete ORF1a sequence (Fig. 3b) and with strain 5 isolates based on recombinant sequence in event-1 (Fig. 3c), but was much closer to strain Pr based on recombinant sequence in event-2 (Fig. 3d). The Estellat isolate of strain 6 formed a separate group in the CP-based phylogenetic tree, but clustered with isolates of strain 5 in trees reconstructed by using the complete ORF1a (Fig. 3b) and recombinant sequence in event-3 (Fig. 3e). Based on the phylogenetic evidence provided in this study, it can be concluded that LR-106 and Estellat isolates were recombinants produced by exchange of genome sequences between distinct strains of GLRaV-4.

## Discussion

Irrespective of minor differences in genome size and nucleotide composition, WAMR-4, WASB-5 and WALA-9 isolates of GLRaV-4 characterized in this study represent, respectively, closely related variants of strain 4, 5, and 9 reported earlier. These three isolates contained six ORFs and their genome organization aligned with other GLRaV-4 strains [9–14]. In addition to absence of the CPm, currently known GLRaV-4 strains differ from other grapevine-infecting ampeloviruses (GLRaV-1, − 3, and − 13) in having smaller size NTRs at both the termini [25]. The long 5′ NTRs of GLRaV-1, − 3, and − 13, varying in size between 672 and 1100 nts, are characterized by having variable number of ~ 65-nt-long repeats [25]. In contrast, GLRaV-4 strains have short 5′ NTR varying in size between 213 and 218 nt without any sequence repeats. Irrespective, a conserved 11-nt sequence is present towards the 5′-terminus in all strains of GLRaV-4. In anology with recently reported functional role for the 5′-terminal conserved sequence in GLRaV-3 [50], it is possible that conserved sequences in the 5′ NTR play a vital role in the life cycle of GLRaV-4 strains. In this regard, a reverse genetic system for GLRaV-4 could provide important clues regarding the functional role of conserved sequences in the 5′ and 3′ NTRs in virus replication and other processes. The availability of infectioucs cDNA clones would also enable confirmation of the extra nucloetides present in some GLRaV-4 isolates beyond the conserved G and C residues, respectively, at the 5′- and 3′-terminus are an integral part of the viral genome.

One would argue that the “mosaic” genomes of LR106 isolate of strain 4 and Estellat isolate of strain 6 were due to fortuitous cross-sequencing of viral mixtures. This is unlikely since the complete genome sequences of two closely related isolates (LR106 and WAMR-4) of strain 4 were obtained independently at different time periods in two geographically separate laboratories (10; this study). Sanger sequencing across the putative recombination junctions further supported sequence continuity in ORF1a of WAMR-4 isolate, thereby discounting errors during amplification and cloning of viral sequences. Additionally, the genome sequences of isolates LR106 and Estellat were generated from distinct grapevine cultivars and unlikely to be cobbled together from portions of other viral sequences during processing of grapevine samples in the laboratory. It is, therefore, reasonable to conclude that mosaic sequences in the genome of isolates LR106 and Estallat are unlikely products of contamination with multiple viral sequences but a consequence of homologous recombination between distinct strains of GLRaV-4.

Several studies have reported genetic variability among closteroviruses, including grapevine-infecting ampleloviruses, driven by both mutations and recombination events [51, 52]. Although nucleotide diversity was reported previously in GLRaV-4 and its strains [52], these analyses were based solely on the CP gene instead of full genome analyses. In contrast, this study using complete genome sequences is the first to show intra-species recombinantion among strains of GLRaV-4, providing strong evidence that LR106 isolate of strain 4 and Estellat isolate of strain 6 are recombinants. The results further suggested that in-frame recombination events in a non-structural protein, such as ORF1a, are nonlethal and could result in the emergence of genetically stable chimeric viruses. Thus, recombination could be an important driver in shaping the genetic diversity and evolution of GLRaV-4.

Although a variety of molecular mechanisms are known to contribute to RNA recombination in positive-strand RNA viruses [53], the molecular basis of recombination events observed in this study are not completely understood and require further studies. It is plausible that recombination in GLRaV-4 isolates occurred via ‘copy-choice’ mechanism due to template switching of the viral RNA polymerase during genome replication. The existence of two distinct breakpoints in isolate LR106 implies two template switches in contrast to one breakpoint in isolate Estellat suggestive of onetime template switching. Eventhough it is difficult to predict when and where the recombination events in GLRaV-4 occurred or the origin of recombinant isolates, a likely scenario would be that co-infection of different strains in grapevines could have increased the probability of producing recombinant isolates and they persisted without being subjected to transmission bottlenecks and disseminated via clonal propagation of planting materials. Promiscuous recombination between multiple, co-replicating strains of CTV infecting citrus was shown to be a major player in promoting the extraordinary diversity of this closterovirus [54]. It is conceivable that similar processes may also be operating with GLRaV-4 strains providing a broader scope of recombination between multiple genotypes within a long-lived perennial host, such as grapevine, and subsequent divergence of these recombinants via clonal propagation and dissemination of infected planting materials. In this context, complete genome analyses of additional isolates from a wide range of grapevine-growing regions are needed to examine the extent of recombination in GLRaV-4 and determine various evolutionary forces shaping genetic diversity of the virus. From a practical point of view, understanding of virus diversification due to recombination will offer insights on epidemiological implications of new variants differing in their biological properties from known strains. Evidently, natural genetic exchange between divergent strains adds a new layer of complexity to the biological understanding of GLRaV 4. Thus, much need to be learned about genome-wide recombination to establish a logical framework for taxonomic separation of prototype strains of GLRaV-4 from recombinants to avoid ambiguity in grouping of ampeloviruses as strains of GLRaV-4 [7].

## Conclusions

In summary, this genome-wide study is the first to show recombinantion among distinct strains of GLRaV-4. Besides providing strong evidence that recombination occurs in natural populations of GLRaV-4, this study also indicates that recombination could play a key role in generating new, biologically successful strains. How recombinant strains of GLRaV-4 have arisen remains a subject for further studies. At the simplest level, full-length sequences of new isolates should be analyzed against well-characterized, full-length sequences of GLRaV-4 strains to determine whether they are recombinants and to avoid misclassification of variant sequences as distinct strains of GLRaV-4. Such comprehensive analyses using full-length sequences is increasingly needed in future to distinguish recombinants from strains arising from other evolutionary processes.

## Additional files

**Additional file 1: Table S1.** Primers used for genome sequencing of GLRaV-4 strains 4, 5, and 9 from Washington vineyards. **Table S2.** Nucleotide and amino acid (in parenthesis) sequence identities of the three isolates (WAMR-4, WASB-5 and WALA-9) of GLRaV-4 strains from Washington vineyards with GLRaV-4 strains from other grapevine-growing regions. **Table S3.** Sequence identities between putative recombinant sequences in strain 4 isolate LR106 and strain 6 isolate Estallat with corresponding sequences in isolates of other GLRaV-4 strains. The columns designated as event-1 and event-2 represent, respectively, nt 4105-5240 and nt 627-1551 in isolate LR106 of strain 4 (accession FJ467503.1). The columns designated as event-3 5’-half and event-3 3’-half represent, respectively, nt 1-6311 and nt 6312-13807 in the genome of isolate Estellat of strain 6 (accession FJ467504.1).

**Additional file 2: Figure S1.** Identification of putative recombination events in GLRaV-4 strains. Graphical representation of (A) the generalized genome map of GLRaV-4 (see description of open reading frames in Fig. 1A) and (B) SimPlot graphs showing nucleotide similarity across the genome of different strains of GLRaV-4. The GLRaV-4 (B1) strain 4 isolate WAMR-4, (B2) strain 5 isolate WASB-5 and (B3) strain 6 isolate Estellat were used as query sequences in respective plots. The X-axis indicates nucleotide position in the alignment and the Y-axis shows percent nucleotide similarity. GenBank accessions of GLRaV-4 used in this analysis were strain 4 isolate LR106 (FJ467503.1), strain 5 isolate 3138–03 (JX559639.1), strain 5 isolate TRAJ1-BR (KX828702.1), strain 6 isolate Estellat (FJ467504.1), strain 9 isolate Man086 (KJ810572.1), strain Pr (AM182328.4) and strain Car (FJ907331.1). Strain Ob (KP313764.1) and strain 5 isolate Y217 from New York (FR822696.2) were not included in the analysis due to the lack of sequence at the 5′ terminus. The color key of the isolates is shown next to the plots.

## Data Availability

The complete genome sequences of WAMR-4 isolate of GLRaV-4 strain 4, WASB-5 isolate of GLRaV-4 strain 5 and WALA-9 isolate of GLRaV-4 strain 9 were deposited in GenBank under the accession number MF669483, MF669481 and MF669482, respectively.

## Abbreviations

aa: Amino acid
AlkB: AlkB domain
bp: Base pair
BYV: Beet yellows virus
cDNA: Complementary DNA
CP: Coat protein
CPm: Minor coat protein
CTV: Citrus tristeza virus
GLRaV: Grapevine leafroll-associated virus
HEL: Helicase
HSP70h: Heat shock protein 70 homologue
HSP90h: Heat shock protein 90 homologue
HTS: High-throughput sequencing
kb: Kilobase
L-Pro: Papain-like leader protease
MEGA7: Molecular evolutionary genetics analysis software
MET: Methyl transferase
nt: Nucleotide
NTR: Non-translated region
ORF: Open reading frame
RACE: Rapid amplification of cDNA ends
RDP: Recombination detection program
RdRp: RNA-dependent RNA polymerase
RGB: Replication gene module
RIN: RNA integrity number
rRNA: Ribosomal RNA
RT-PCR: Reverse transcription-polymerase chain reaction
sgRNA: Subgenomic RNA

## References

[1] Naidu RA, Maree HJ, Burger JT (2015). Grapevine leafroll disease and associated viruses: a unique Pathosystem. Annu Rev Phytopathol.

[2] Dolja VV, Meng B, Martelli GP, Meng B, Martelli GP, Golino D (2017). Evolutionary aspects of grapevine virology. Grapevine viruses: molecular biology, diagnostics and management.

[3] Herrbach E, Alliaume A, Prator C, Daane K, Cooper M, Almeida R, Meng B, Martelli GP, Golino D (2017). Vector transmission of grapevine leafroll-associated viruses. Grapevine viruses: molecular biology, diagnostics and management.

[4] Mikona C, Jelkmann W (2010). Replication of *Grapevine leafroll-associated virus*-7 (GLRaV-7) by *Cuscuta* species and its transmission to herbaceous plants. Plant Dis.

[5] Maliogka VI, Dovas CI, Katis NI (2008). Evolutionary relationships of virus species belonging to a distinct lineage within the *Ampelovirus* genus. Virus Res.

[6] Martelli GP, Abou Ghanem-Sabanadzovic N, Agranovsky AA, Al Rwahnih M, Dolja VV, Dovas CI (2012). Taxonomic revision of the family *Closteroviridae* with special reference to the grapevine leafroll-associated members of the genus *Ampelovirus* and the putative species unassigned to the family. J Plant Pathol.

[7] Abou Ghanem-Sabanadzovic N, Maglioka V, Sabanadzovic S, Meng B, Martelli GP, Golino D (2017). Grapevine leafroll-associated virus 4. Grapevine viruses: molecular biology, diagnostics and management.

[8] Ito T, Nakaune R, Nakano M, Suzaki K (2013). Novel variants of grapevine leafroll-associated virus 4 and 7 detected from a grapevine showing leafroll symptoms. Arch Virol.

[9] Reynard JS, Schneeberger PHH, Frey JE, Schaerer S (2015). Biological, serological, and molecular characterization of a highly divergent strain of *Grapevine leafroll-associated virus* 4 causing grapevine leafroll disease. Phytopathology..

[10] Abou Ghanem-Sabanadzovic N, Sabanadzovic S, Gugerli P, Rowhani A (2012). Genome organization, serology and phylogeny of grapevine leafroll-associated viruses 4 and 6: taxonomic implications. Virus Res.

[11] Maliogka VI, Dovas CI, Lotos L, Efthimiou K, Katis NI (2009). Complete genome analysis and immunodetection of a member of a novel virus species belonging to the genus *Ampelovirus*. Arch Virol.

[12] Abou Ghanem-Sabanadzovic N, Sabanadzovic S, Uyemoto JK, Golino D, Rowhani A (2010). A putative new ampelovirus associated with grapevine leafroll disease. Arch Virol.

[13] Velasco L, Cretazzo E, Padilla CV, Janssen D (2015). Grapevine leafroll associated virus 4 strain 9: complete genome and quantitative analysis of virus-derived small interfering RNA populations. J Plant Pathol.

[14] Thompson JR, Fuchs M, Perry KL (2012). Genomic analysis of *Grapevine leafroll associated virus*-5 and related viruses. Virus Res.

[15] Golino D, Sim S, Gill R, Rowhani A (2002). California mealybugs can spread grapevine leafroll disease. Calif Agric.

[16] Sharma AM, Baraff B, Hutchins JT, Wong MK, Blaisdell GK, Cooper ML (2015). Relative prevalence of grapevine leafroll-associated virus species in wine grape-growing regions of California. PLoS One.

[17] Bahder BW, Alabi O, Poojari S, Walsh DB, Naidu RA (2013). A survey for grapevine viruses in Washington State ‘Concord’(*Vitis* × *labruscana* L.) vineyards. Plant Health Prog.

[18] Jarugula S, Soule MJ, Rowhani A, Naidu RA (2008). First report of *Grapevine leafroll-associated virus*-9 in Washington state vineyards. Plant Dis.

[19] Mekuria TA, Soule MJ, Jarugula S, Naidu RA (2009). Current status of grapevine viruses in Washington state vineyards. Phytopathology.

[20] Naidu RA. Virus update: The status of Washington vineyards. In: Viticulture and Enology Extension News-Fall 2011: Washington State University. p. 6–7. https://research.libraries.wsu.edu:8443/xmlui/bitstream/handle/2376/13461/VEEN-Fall2011.pdf?sequence=1&isAllowed=y

[21] Alabi OJ, Martin RR, Naidu RA (2010). Sequence diversity, population genetics and potential recombination events in grapevine rupestris stem pitting-associated virus in Pacific north-west vineyards. J Gen Virol.

[22] Jones TJ, Rayapati NA, Nita M (2015). Occurrence of *Grapevine leafroll associated virus*-2, −3 and *Grapevine fleck virus* in Virginia, USA, and factors affecting virus infected vines. Eur J Plant Pathol.

[23] Osman F, Leutenegger C, Golino D, Rowhani A (2007). Real-time RT-PCR (TaqMan (R)) assays for the detection of *Grapevine leafroll associated viruses* 1-5 and 9. J Virol Methods.

[24] Alkowni R, Rowhani A, Daubert S, Golino D (2004). Partial characterization of a new ampelovirus associated with grapevine leafroll disease. J Plant Pathol.

[25] Donda BP, Jarugula S, Naidu RA (2017). An analysis of the complete genome sequence and subgenomic RNAs reveals unique features of the Ampelovirus, *Grapevine leafroll-associated virus* 1. Phytopathology.

[26] Jarugula Sridhar, Gowda Siddarame, Dawson William O, Naidu Rayapati A (2010). 3'-coterminal subgenomic RNAs and putative cis-acting elements of Grapevine leafroll-associated virus 3 reveals 'unique' features of gene expression strategy in the genus Ampelovirus. Virology Journal.

[27] Edgar RC (2004). MUSCLE: a multiple sequence alignment method with reduced time and space complexity. BMC Bioinformatics.

[28] Kumar S, Stecher G, Tamura K (2016). MEGA7: molecular evolutionary genetics analysis version 7.0 for bigger datasets. Mol Biol Evol.

[29] Lole KS, Bollinger RC, Paranjape RS, Gadkari D, Kulkarni SS, Novak NG (1999). Full-length human immunodeficiency virus type 1 genomes from subtype C-infected seroconverters in India, with evidence of intersubtype recombination. J Virol.

[30] Felsenstein J (1981). Evolutionary trees from gene-frequencies and quantitative characters - finding maximum-likelihood estimates. Evolution.

[31] Martin DP, Murrell B, Golden M, Khoosal A, Muhire B. RDP4: Detection and analysis of recombination patterns in virus genomes. Virus Evol. 2015;1(1):vev003. 10.1093/ve/vev003 PMC5014473 27774277

[32] Peng CW, Peremyslov VV, Mushegian AR, Dawson WO, Dolja VV (2001). Functional specialization and evolution of leader proteinases in the family *Closteroviridae*. J Virol.

[33] Finn RD, Bateman A, Clements J, Coggill P, Eberhardt RY, Eddy SR (2014). Pfam: the protein families database. Nucleic Acids Res.

[34] van den Born E, Omelchenko MV, Bekkelund A, Leihne V, Koonin EV, Dolja VV (2008). Viral AlkB proteins repair RNA damage by oxidative demethylation. Nucleic Acids Res.

[35] Agranovsky AA, Koonin EV, Boyko VP, Maiss E, Frotschl R, Lunina NA (1994). Beet yellows closterovirus - complete genome structure and identification of a leader papain-like thiol protease. Virology.

[36] Fazeli CF, Rezaian MA (2000). Nucleotide sequence and organization of ten open reading frames in the genome of grapevine leafroll-associated virus 1 and identification of three subgenomic RNAs. J Gen Virol.

[37] Ling KS, Zhu HY, Gonsalves D (2004). Complete nucleotide sequence and genome organization of *Grapevine leafroll-associated virus* 3, type member of the genus *Ampelovirus*. J Gen Virol.

[38] Rott ME, Jelkmann W (2005). Little cherry virus-2: sequence and genomic organization of an unusual member of the *Closteroviridae*. Arch Virol.

[39] Thekke-Veetil T, Aboughanem-Sabanadzovic N, Keller KE, Martin RR, Sabanadzovic S, Tzanetakis IE (2013). Molecular characterization and population structure of blackberry vein banding associated virus, new ampelovirus associated with yellow vein disease. Virus Res.

[40] Koonin EV, Dolja VV, Morris TJ (1993). Evolution and taxonomy of positive-strand RNA viruses: implications of comparative analysis of amino acid sequences. Crit Rev Biochem Mol Biol.

[41] Kiss ZA, Medina V, Falk BW. *Crinivirus* replication and host interactions. Front Microbiol. 2013;4:99. 10.3389/fmicb.2013.00099 PMC3657685 23730299

[42] Napuli AJ, Alzhanova DV, Doneanu CE, Barofsky DF, Koonin EV, Dolja VV (2003). The 64-kilodalton capsid protein homolog of *Beet yellows virus* is required for assembly of virion tails. J Virol.

[43] He XH, Rao ALN, Creamer R (1997). Characterization of beet yellows closterovirus-specific RNAs in infected plants and protoplasts. Phytopathology.

[44] Peremyslov VV, Dolja VV (2002). Identification of the subgenomic mRNAs that encode 6-kDa movement protein and hsp70 homolog of *Beet yellows virus*. Virology..

[45] Dawson WO. Molecular genetics of Citrus tristeza virus. In: *Citrus tristeza virus* complex and tristeza diseases: APS Press St. Paul; 2010. p. 53–72.

[46] Chiba M, Reed JC, Prokhnevsky AI, Chapman EJ, Mawassi M, Koonin EV (2006). Diverse suppressors of RNA silencing enhance agroinfection by a viral replicon. Virology.

[47] Lu R, Folimonov A, Shintaku M, Li WX, Falk BW, Dawson WO (2004). Three distinct suppressors of RNA silencing encoded by a 20-kb viral RNA genome. Proc Natl Acad Sci U S A.

[48] Prokhnevsky AI, Peremyslov VV, Napuli AJ, Dolja VV (2002). Interaction between long-distance transport factor and Hsp70-related movement protein of *Beet yellows virus*. J Virol.

[49] Reed JC, Kasschau KD, Prokhnevsky AI, Gopinath K, Pogue GP, Carrington JC (2003). Suppressor of RNA silencing encoded by *Beet yellows virus*. Virology.

[50] Jarugula S, Gowda S, Dawson WO, Naidu RA (2018). Development of infectious cDNA clones of *Grapevine leafroll-associated virus* 3 and analyses of the 5 ' non-translated region for replication and virion formation. Virology.

[51] Maree Hans J., Pirie Michael D., Oosthuizen Kristin, Bester Rachelle, Rees D. Jasper G., Burger Johan T. (2015). Phylogenomic Analysis Reveals Deep Divergence and Recombination in an Economically Important Grapevine Virus. PLOS ONE.

[52] Rubio L, Guerri J, Moreno P. Genetic variability and evolutionary dynamics of viruses of the family *Closteroviridae*. Front Microbiol. 2013;4,151. 10.3389/fmicb.2013.00151 PMC3693128 23805130

[53] Bujarski JJ. Genetic recombination in plant-infecting messenger-sense RNA viruses: overview and research perspectives. Front Plant Sci. 2013;4,68. 10.3389/fpls.2013.00068 PMC3607795 23533000

[54] Martín S, Sambade A, Rubio L, Vives MC, Moya P, Guerri J, Elena SF, Moreno P (2009). Contribution of recombination and selection to molecular evolution of *Citrus tristeza virus*. J Gen Virol.

