# Supplementary material for: Intra-species recombination among strains of the ampelovirus Grapevine leafroll-associated virus 4
Source: Virol J. 2019 Nov 19;16:139. doi: 10.1186/s12985-019-1243-4 (PMC6862812; doi:10.1186/s12985-019-1243-4)
Supplement: Supplementary file 1 — Additional file 1: Table S1. Primers used for genome sequencing of GLRaV-4 strains 4, 5, and 9 from Washington vineyards. Table S2. Nucleotide and amino acid (in parenthesis) sequence identities of the three isolates (WAMR-4, WASB-5 and WALA-9) of GLRaV-4 strains from Washington vineyards with GLRaV-4 strains from other grapevine-growing regions. Table S3. Sequence identities between putative recombinant sequences in strain 4 isolate LR106 and strain 6 isolate Estallat with corresponding sequences in isolates of other GLRaV-4 strains. The columns designated as event-1 and event-2 represent, respectively, nt 4105-5240 and nt 627-1551 in isolate LR106 of strain 4 (accession FJ467503.1). The columns designated as event-3 5’-half and event-3 3’-half represent, respectively, nt 1-6311 and nt 6312-13807 in the genome of isolate Estellat of strain 6 (accession FJ467504.1). [file 12985_2019_1243_MOESM1_ESM.docx]

**Table S1**. Primers used for genome sequencing of GLRaV-4 strains 4, 5, and 9 from Washington vineyards.

| Primer name | Primer sequence (5'-3') | Coordinates | Description |
| --- | --- | --- | --- |
| LR4-454F | GAAAGGGTAAGCAAGCCACTGAC | 454-477* | HTS confirmation |
| LR4-1600R | CCTGTTGGGTATTGTGTTAACGGT | 1577-1600* | HTS confirmation |
| LR4-3952F | TGGAGAAGGTGATGAGTGAGCTC | 3952-3974* | HTS confirmation |
| LR4-5414R | TAAGGTATTCCGGCCCCACACT | 5389-5410* | HTS confirmation |
| LR4-13072F | GATATGGAGGTTATTCTTTGC | 13072-13103* | HTS confirmation |
| LR4-3'R | GGTCCTGGATCTCTCCAAG | 13810-13830* | HTS confirmation |
| LR4-5'F | GTAATCTTTTGCTAGGGCTATC | 1-22* | HTS confirmation |
| LR4-262R | CTGCAAGTGGAGGGTCGTC | 262-381* | HTS/ 5' RACE GSP1 |
| LR4-157R | GAAAGCACTCAAAGCGAGAC | 157-177* | 5' RACE GSP2 |
| LR4-13604F | AGTAACACGGCGCTTCTCGAGT | 13604-13622* | 3' RACE GSP1 |
| LR4-13642F | CTCTGCTTCGGGAAAACCAGAT | 13642-13666* | 3' RACE GSP2 |
| LR9-12976F | GTGGCCATCAATAAACCATCC | 12976-12996** | HTS confirmation |
| LR9-3PR | CGGTTCCTGGATTTCTCCAAG | 13879-13858** | HTS confirmation |
| LR9-3286F | GACGTTGCTGTCCGCACCAG | 3286-3310** | HTS confirmation |
| LR9-3688R | CCTTGCTGCAGTGGTGACCG | 3669-3688** | HTS confirmation |
| LR9-5PF | AACGTAATCTTTTGCTAGGGC | 1 to 20** | HTS confirmation |
| LR9-2714R | GGATTTGTTCCTCAACCAGG | 2714-2734** | HTS confirmation |
| LR9-4784F | TAAGGCTAGAATACCCAGTCCCGCT | 4784-4807** | HTS confirmation |
| LR9-5112R | AGGAGTCTGAGACTGTGTCGGCGAT | 5112-5137** | HTS confirmation |
| LR9-13515F | AGTGTTAGCGGTCGGTTTCAC | 13515-1535** | 3' RACE GSP1 |
| LR9-13649F | CGCTTCTCGAAGTTTTAGAC | 13649-13668** | 3' RACE GSP2 |
| LR9-237R | AGACGGACGGTTGAACATGCT | 237-258** | 5' RACE GSP1 |
| LR9-176R | GGTGGATACTCACAAAGAGAG | 176-187** | 5' RACE GSP2 |
| LR5-642R | GCCCAGAAGAAAGGGAGCTGT | 642-665*** | HTS confirmation |
| LR5-5PF | MCGTAATCTTTTGCTAGGGCTATCC | 1-25*** | HTS confirmation |
| LR5-3P | CGGTTCCTGGATTTCTCCAAG | 13801-13823*** | HTS confirmation |
| LR5-13010F | GACATCTTCCTGGGTAACAGGA | 13010-13023*** | HTS confirmation |
| LR5-13462F | TGAGGGGCTAACAACGGTGTTA | 13462-13484*** | 3' RACE GSP1 |
| LR5-13595F | GACACCAGTAATTCGGCGCTTCT | 13595-13618*** | 3' RACE GSP2 |
| M111a | GGTCTCGAG(T)18 |  | RACE |

*Based on WAMR-4, ** Based on WALA-9, *** Based on WASB-5

**Table S2.** Nucleotide and amino acid (in parenthesis) sequence identities of the three isolates (WAMR4, WASB5 and WALA9) of GLRaV-4 strains from Washington vineyards with GLRaV-4 strains from other grapevine-growing regions.

|  | genome | | | 5′ NTR | | | ORF1a | | | ORF1b | | | p5 | | |
| --- | --- | --- | --- | --- | --- | --- | --- | --- | --- | --- | --- | --- | --- | --- | --- |
|  | **WA**  **MR4** | **WA**  **SB5** | **WA**  **LA9** | **WA**  **MR4** | **WA**  **SB5** | **WA**  **LA9** | **WA**  **MR4** | **WA**  **SB5** | **WA**  **LA9** | **WA**  **MR4** | **WA**  **SB5** | **WA**  **LA9** | **WA**  **MR4** | **WA**  **SB5** | **WA**  **LA9** |
| St 4_WAMR4 | 100.0 | 62.6 | 61.6 | 100.0 | 61.4 | 61.0 | 100 (100) | 62.6 (57.2) | 61.6 (55.5) | 100 (100) | 75.2 (89) | 74.7 (90.7) | 100 (100) | 74.4 (83.5) | 75.9 (93.3) |
| St 4_LR106 | 93.2 | 66.1 | 62.2 | 96.8 | 62.6 | 59.5 | 93.2 (81.8) | 66.1 (64.5) | 62.2 (57.6) | 96.8 (95.9) | 73.1 (85) | 72.8 (86.6) | 98.6 (97.8) | 76.5 (86) | 73.6 (90.9) |
| St 5_WASB5 | 62.6 | 100.0 | 70.4 | 61.4 | 100.0 | 73.9 | 62.6 (57.2) | 100 (100) | 70.4 (69.2) | 75.2 (89) | 100 (100) | 77.6 (91.7) | 74.4 (83.5) | 100 (100) | 78.4 (86) |
| St 5_3138-03 | 61.8 | 93.3 | 70.2 | 60.4 | 95.2 | 75.3 | 61.8 (57.2) | 93.3 (92.2) | 70.2 (68.4) | 74.2 (88.5) | 95 (97.8) | 77.4 (91.3) | 68.7 (90.9) | 94.1 (93.3) | 82.3 (93.3) |
| St 5_TRAJ1 | 62.0 | 93.4 | 70.2 | 64.9 | 93.5 | 77.7 | 62.0 (57.0) | 93.4 (92.5) | 70.2 (68.9) | 74 (89.8) | 94.8 (97.3) | 77.1 (91.5) | 76.3 (90.9) | 94.8 (90.9) | 82.2 (90.9) |
| St 5_Y217 | 61.9 | 93.3 | 70.4 | 28.7 | 46.9 | 41.5 | 61.9 (57.8) | 93.3 (92.6) | 70.4 (68.2) | 74 (88.5) | 95 (97.8) | 77.3 (91.3) | 70.7 (90.9) | 93.3 (93.3) | 84.1 (93.3) |
| St 6_Estellat | 60.9 | 81.0 | 67.6 | 64.9 | 92.9 | 78.3 | 60.9 (55.5) | 81 (90.1) | 67.6 (67.7) | 72.1 (89.4) | 74.1 (89.6) | 73.7 (90.7) | 73 (88.5) | 80.7 (86) | 76.5 (88.5) |
| St 9_WALA9 | 61.6 | 70.4 | 100.0 | 61.0 | 73.9 | 100.0 | 61.6 (55.5) | 70.4 (69.2) | 100 (100) | 74.7 (90.7) | 77.6 (91.7) | 100 (100) | 75.9 (93.3) | 78.4 (86) | 100 (100) |
| St 9_Man086 | 61.2 | 70.7 | 93.8 | 64.9 | 83.9 | 84.9 | 61.2 (55.1) | 70.7 (68.9) | 93.8 (92.8) | 75.5 (90.3) | 77.9 (91.7) | 95.8 (98.8) | 74.8 (93.3) | 75.2 (83.5) | 95.6 (97.8) |
| St Car | 42.3 | 46.3 | 42.4 | 39.4 | 27.1 | 8.1 | 42.3 (25.9) | 46.3 (26) | 42.4 (22.1) | 58.1 (73) | 58.4 (72.3) | 58.5 (74) | 67.6 (75.5) | 65.8 (63.7) | 63.8 (72.7) |
| St Ob | 45.3 | 48.1 | 45.3 | 16.8 | 22.9 | 40.4 | 45.3 (28.5) | 48.1 (27.7) | 45.3 (28.3) | 59.4 (73.8) | 59.7 (74.5) | 58.7 (73) | 57.4 (75.5) | 64.8 (69.8) | 65.8 (78.2) |
| St Pr | 53.7 | 54.4 | 52.3 | 63.9 | 62.1 | 61.2 | 53.7 (40.8) | 54.4 (41.6) | 52.3 (39.5) | 65.8 (82.7) | 68 (81.1) | 65.6 (82.3) | 61.1 (75.5) | 62.2 (80.9) | 64.6 (80.9) |

|  | HSP70h | | | p60 | | | CP | | | p23 | | | 3′ NTR | | |
| --- | --- | --- | --- | --- | --- | --- | --- | --- | --- | --- | --- | --- | --- | --- | --- |
|  | **WA**  **MR4** | **WA**  **SB5** | **WA**  **LA9** | **WA**  **MR4** | **WA**  **SB5** | **WA**  **LA9** | **WA**  **MR4** | **WA**  **SB5** | **WA**  **LA9** | **WA**  **MR4** | **WA**  **SB5** | **WA**  **LA9** | **WA**  **MR4** | **WA**  **SB5** | **WA**  **LA9** |
| St 4_WAMR4 | 100 (100) | 67 (80.4) | 67 (80.4) | 100 (100) | 65.6 (79.9) | 66.2 (79.3) | 100 (100) | 67.5 (82.4) | 69 (83.3) | 100 (100) | 75.7 (65.1) | 75.7 (65.1) | 100.0 | 90.8 | 95.0 |
| St 4_LR106 | 99.3 (99.6) | 66.9 (80.4) | 67.2 (80.4) | 98.8 (98.7) | 64.7 (78.6) | 65.8 (77.9) | 99.7 (99.6) | 67.5 (82.8) | 69 (83.7) | 99.4 (98.5) | 75.7 (65.8) | 75.6 (65.8) | 99.2 | 89.8 | 94.1 |
| St 5_WASB5 | 67 (80.4) | 100 (100) | 74.6 (88.9) | 65.6 (79.9) | 100 (100) | 74.1 (86.9) | 67.5 (82.4) | 100 (100) | 75.4 (85.6) | 75.7 (65.1) | 100 (100) | 88.8 (89.3) | 90.8 | 100.0 | 92.3 |
| St 5_3138-03 | 67.5 (80.4) | 94.1 (97.3) | 75.1 (89.1) | 64.2 (78.1) | 94.6 (97) | 74.5 (86.9) | 64.9 (82.4) | 93.6 (96.6) | 77  (86) | 75.3 (65.1) | 96.7 (95.6) | 89.2 (90.9) | 91.7 | 99.2 | 93.2 |
| St 5_TRAJ1 | 66.7 (80.1) | 93.7 (96.6) | 73.6 (87.8) | 63.9 (77.6) | 94.6 (95.6) | 74.2 (84.8) | 66.9 (83.7) | 93.9 (95.4) | 75.6 (85.1) | 76.1 (67.1) | 96.5 (97.1) | 87.8 (89.8) | 91.7 | 99.2 | 93.2 |
| St 5_Y217 | 67.1 (80.1) | 93.9 (97.7) | 75.2 (89.1) | 64.3 (78.1) | 94.7 (97.2) | 74.3 (86.5) | 64.9 (82.4) | 93.6 (96.6) | 77.4 (86) | 75.5 (65.1) | 96.9 (95.6) | 89.4 (90.9) | 92.5 | 98.4 | 94.1 |
| St 6_Estellat | 64.1 (75.5) | 69.1 (84.2) | 69.4 (82) | 63.2 (75.5) | 68.5 (79) | 69.5 (79.5) | 66.3 (79.6) | 71.3 (83.8) | 72.8 (83.4) | 71 (65.1) | 82.7 (80.3) | 83.5 (84.3) | 94.3 | 85.2 | 92.4 |
| St 9_WALA9 | 67 (80.4) | 74.6 (88.9) | 100 (100) | 66.2 (79.3) | 74.1 (86.9) | 100 (100) | 69 (83.3) | 75.4 (85.6) | 100 (100) | 75.7 (65.1) | 88.8 (89.3) | 100 (100) | 95.0 | 92.3 | 100.0 |
| St 9_Man086 | 65.9 (80.1) | 75.1 (88.7) | 95.4 (95.6) | 66.9 (79) | 74.7 (86.7) | 94.5 (95.6) | 67.4 (82.4) | 75.8 (86.4) | 96.3 (97.7) | 74.1 (63) | 90.9 (90.9) | 96.2 (96.1) | 93.2 | 92.3 | 98.4 |
| St Car | 45.4 (59.6) | 46.7 (60.2) | 46.3 (59.9) | 43 (57.4) | 44.7 (55.4) | 43.2 (56.2) | 61.6 (76.6) | 63.3 (75.4) | 61.2 (75.9) | 43 (36.8) | 45.2 (41.2) | 44.8 (42.1) | 84.0 | 83.0 | 86.6 |
| St Ob | 51.1 (65.1) | 50.5 (62.7) | 51.5 (64.3) | 45.8 (55.1) | 45.3 (56.5) | 45 (56.8) | 64.7 (75.6) | 63.3 (76.3) | 58.5 (72.4) | 46.3 (37.7) | 45.4 (36.8) | 42.1 (37.7) | 85.2 | 83.1 | 86.8 |
| St Pr | 60.7 (74.7) | 59.3 (77.6) | 61.1 (77.8) | 54.5 (66.4) | 55.5 (68.9) | 52.8 (66.9) | 61.3 (76.8) | 60.6 (75.1) | 57.9 (75.6) | 59.3 (53.5) | 60.4 (52.7) | 57.9 (53.5) | 89.8 | 89.0 | 85.7 |

**Table S3.** Sequence identities between putative recombinant sequences in strain 4 isolate LR106 and strain 6 isolate Estallat with corresponding sequences in isolates of other GLRaV-4 strains. The columns designated as event-1 and event-2 represent, respectively, nt 4105-5240 and nt 627-1551 in isolate LR106 of strain 4 (accession FJ467503.1). The columns designated as event-3 5’-half and event-3 3’-half represent, respectively, nt 1-6311 and nt 6312-13807 in the genome of isolate Estellat of strain 6 (accession FJ467504.1).

|  | Event-1 | Event-2 | Event-3 5' half | Event-3 3' half |
| --- | --- | --- | --- | --- |
| St 4_WAMR4 | 38.24 | 47.87 | 52.03 | 67.44 |
| St 4_LR106 | 100.00 | 100.00 | 61.05 | 67.37 |
| St 5_WASB5 | 90.31 | 50.75 | 90.74 | 71.96 |
| St 5_3138-03 | 99.17 | 48.33 | 90.03 | 71.12 |
| St 5_TRAJ1-BR | 88.32 | 49.38 | 89.83 | 71.32 |
| St 5_Y217 | 98.43 | 49.72 | 89.97 | 71.08 |
| St 6_Estellat | 89.11 | 49.35 | 100.00 | 100.00 |
| St 9_WALA9 | 48.85 | 46.33 | 61.41 | 72.38 |
| St 9_Man086 | 49.14 | 47.09 | 61.72 | 72.48 |
| St Car | 20.06 | 40.66 | 35.81 | 52.83 |
| St Ob | 25.02 | 32.96 | 35.80 | 52.66 |
| St Pr | 23.84 | 88.56 | 43.78 | 61.48 |
