# Supplementary material for: Intra-species recombination among strains of the ampelovirus Grapevine leafroll-associated virus 4
Source: Virol J. 2019 Nov 19;16:139. doi: 10.1186/s12985-019-1243-4 (PMC6862812; doi:10.1186/s12985-019-1243-4)
Supplement: Supplementary file 2 — Additional file 2: Figure S1. Identification of putative recombination events in GLRaV-4 strains. Graphical representation of (A) the generalized genome map of GLRaV-4 (see description of open reading frames in Fig. 1A) and (B) SimPlot graphs showing nucleotide similarity across the genome of different strains of GLRaV-4. The GLRaV-4 (B1) strain 4 isolate WAMR-4, (B2) strain 5 isolate WASB-5 and (B3) strain 6 isolate Estellat were used as query sequences in respective plots. The X-axis indicates nucleotide position in the alignment and the Y-axis shows percent nucleotide similarity. GenBank accessions of GLRaV-4 used in this analysis were strain 4 isolate LR106 (FJ467503.1), strain 5 isolate 3138–03 (JX559639.1), strain 5 isolate TRAJ1-BR (KX828702.1), strain 6 isolate Estellat (FJ467504.1), strain 9 isolate Man086 (KJ810572.1), strain Pr (AM182328.4) and strain Car (FJ907331.1). Strain Ob (KP313764.1) and strain 5 isolate Y217 from New York (FR822696.2) were not included in the analysis due to the lack of sequence at the 5′ terminus. The color key of the isolates is shown next to the plots. [file 12985_2019_1243_MOESM2_ESM.pptx]

## Slide 1
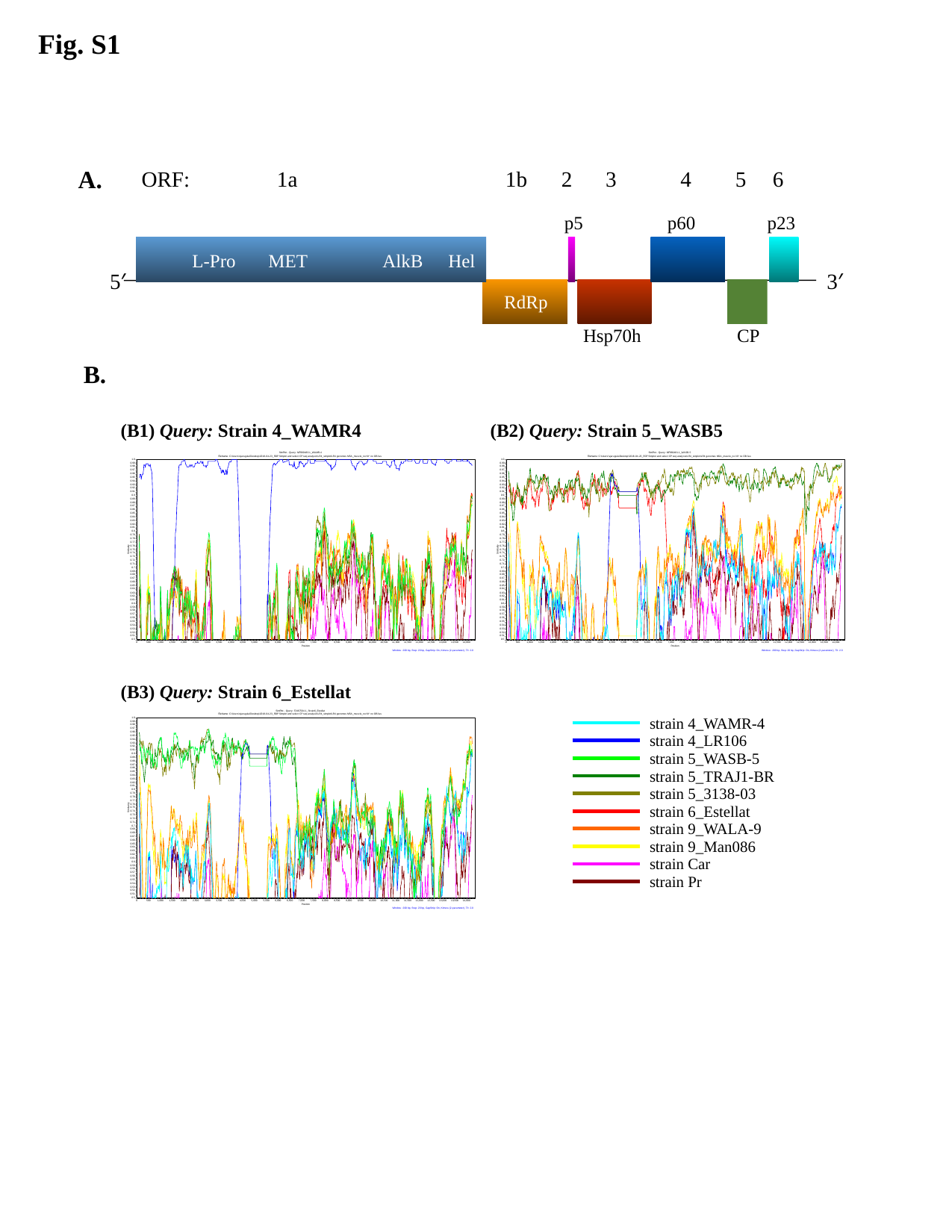

Fig. S1
A.
ORF:
1a
1b
2
3
4
5
6
p5
p60
p23
L-Pro
MET
AlkB
Hel
5′
3′
RdRp
Hsp70h
CP
B.
(B1) Query: Strain 4_WAMR4
(B2) Query: Strain 5_WASB5
(B3) Query: Strain 6_Estellat
strain 4_WAMR-4
strain 4_LR106
strain 5_WASB-5
strain 5_TRAJ1-BR
strain 5_3138-03
strain 6_Estellat
strain 9_WALA-9
strain 9_Man086
strain Car
strain Pr

## Slide 2
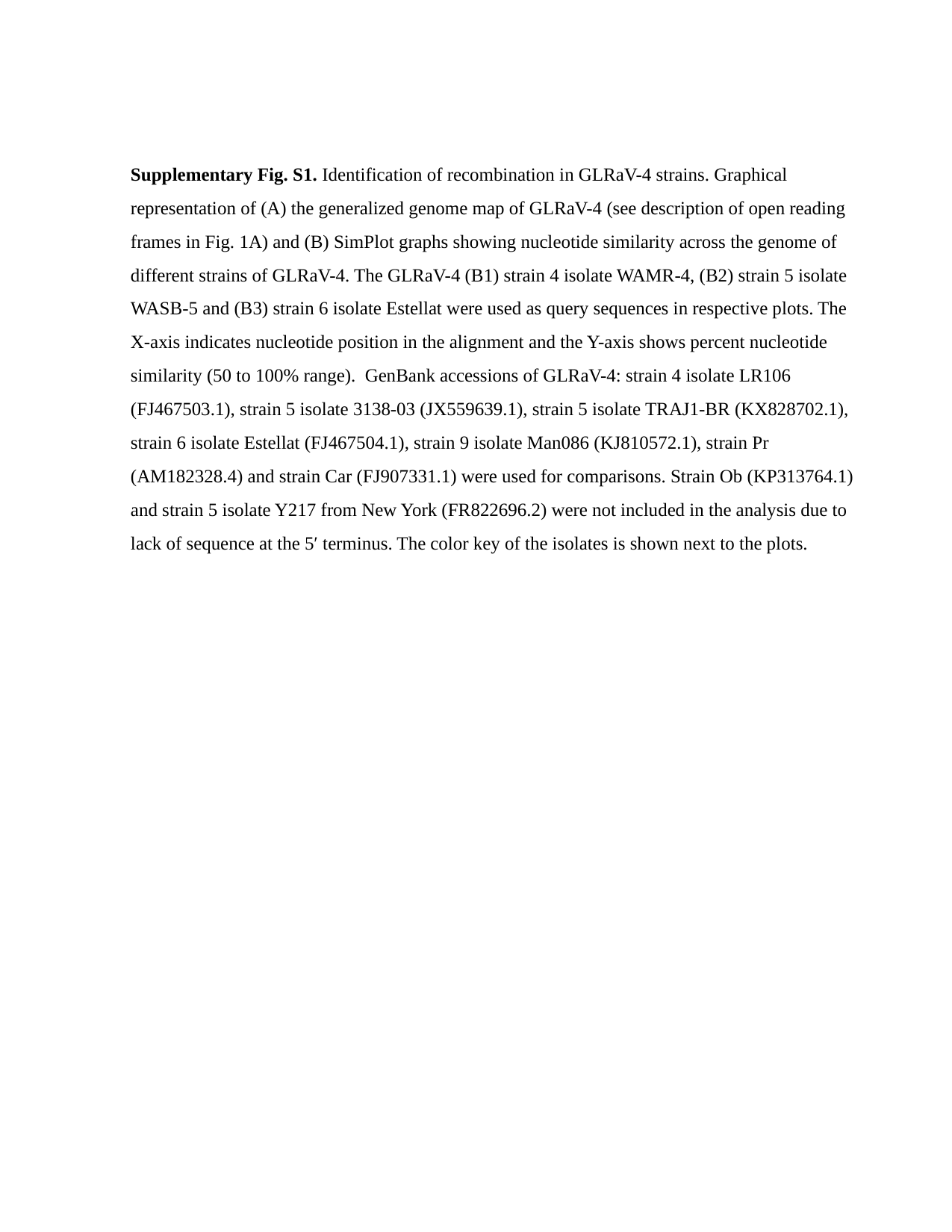

Supplementary Fig. S1. Identification of recombination in GLRaV-4 strains. Graphical representation of (A) the generalized genome map of GLRaV-4 (see description of open reading frames in Fig. 1A) and (B) SimPlot graphs showing nucleotide similarity across the genome of different strains of GLRaV-4. The GLRaV-4 (B1) strain 4 isolate WAMR-4, (B2) strain 5 isolate WASB-5 and (B3) strain 6 isolate Estellat were used as query sequences in respective plots. The X-axis indicates nucleotide position in the alignment and the Y-axis shows percent nucleotide similarity (50 to 100% range). GenBank accessions of GLRaV-4: strain 4 isolate LR106 (FJ467503.1), strain 5 isolate 3138-03 (JX559639.1), strain 5 isolate TRAJ1-BR (KX828702.1), strain 6 isolate Estellat (FJ467504.1), strain 9 isolate Man086 (KJ810572.1), strain Pr (AM182328.4) and strain Car (FJ907331.1) were used for comparisons. Strain Ob (KP313764.1) and strain 5 isolate Y217 from New York (FR822696.2) were not included in the analysis due to lack of sequence at the 5′ terminus. The color key of the isolates is shown next to the plots.
